# Characterization of Osteogenesis and Chondrogenesis of Human Decellularized Allogeneic Bone with Mesenchymal Stem Cells Derived from Bone Marrow, Adipose Tissue, and Wharton’s Jelly

**DOI:** 10.3390/ijms22168987

**Published:** 2021-08-20

**Authors:** Cheng-Fong Chen, Yi-Chun Chen, Yu-Show Fu, Shang-Wen Tsai, Po-Kuei Wu, Chao-Ming Chen, Ming-Chau Chang, Wei-Ming Chen

**Affiliations:** 1 Department of Orthopaedics and Traumatology, Taipei Veterans General Hospital, Taipei 11217, Taiwan; cf_chen@vghtpe.gov.tw (C.-F.C.); swtsai.vghtpe@gmail.com (S.-W.T.); drwuvgh@gmail.com (P.-K.W.); excelnova@gmail.com (C.-M.C.); mcchang@vghtpe.gov.tw (M.-C.C.); wmchen@vghtpe.gov.tw (W.-M.C.); 2 Department of Orthopaedics, School of Medicine, National Yang Ming Chiao Tung University, Taipei 11221, Taiwan; 3 Department of Anatomy and Cell Biology, Faculty of Medicine, National Yang Ming Chiao Tung University, Taipei 11221, Taiwan; ysfu@nycu.edu.tw

**Keywords:** MSC, osteogenesis, chondrogenesis, allogeneic bone

## Abstract

Allogeneic bone grafts are a promising material for bone implantation due to reduced operative trauma, reduced blood loss, and no donor-site morbidity. Although human decellularized allogeneic bone (hDCB) can be used to fill bone defects, the research of revitalizing hDCB blocks with human mesenchymal stem cells (hMSCs) for osteochondral regeneration is missing. The hMSCs derived from bone marrow, adipose tissue, and Wharton’s jelly (BMMSCs, ADMSCs, and UMSCs, respectively) are potential candidates for bone regeneration. This study characterized the potential of hDCB as a scaffold for osteogenesis and chondrogenesis of BMMSCs, ADMSCs, and UMSCs. The pore sizes and mechanical strength of hDCB were characterized. Cell survival and adhesion of hMSCs were investigated using MTT assay and F-actin staining. Alizarin Red S and Safranin O staining were conducted to demonstrate calcium deposition and proteoglycan production of hMSCs after osteogenic and chondrogenic differentiation, respectively. A RT-qPCR was performed to analyze the expression levels of osteogenic and chondrogenic markers in hMSCs. Results indicated that BMMSCs and ADMSCs exhibited higher osteogenic potential than UMSCs. Furthermore, ADMSCs and UMSCs had higher chondrogenic potential than BMMSCs. This study demonstrated that chondrogenic ADMSCs- or UMSCs-seeded hDCB might be potential osteochondral constructs for osteochondral regeneration.

## 1. Introduction

An osteochondral lesion is a localized area of damage, which involves the cartilage and the underlying bone. Mosaicplasty or autologous chondrocyte implantation (ACI) is the standard treatment for osteochondral defects. Filling large osteochondral defects using an osteochondral autograft transfer system (OATS) was excellent in the short term. However, technical difficulty, donor-site morbidity, and poor integration of OATS or mosaicplasty present significant challenges to orthopedic surgeons [1]. Finding alternative therapeutic methods for osteochondral defects is essential.

Decellularized bone matrixes from xenografts or allografts as alternatives have been intensively studied. They can be particles with hydrogels and the original forms revitalized with the help of MSCs [2]. Bone allograft is a mineralized biomaterial that can be harvested from cadavers or patients with replacement surgery and can be decellularized to serve as a scaffold, which preserves the natural microarchitecture, extracellular matrix (ECM), bioactive molecules, and mechanical properties. Furthermore, removing cells and DNA can prevent immune responses after implantation [3].

Although ACI is the standard treatment of osteochondral defects, rapid dedifferentiation of chondrocytes is the drawback when expanded in vitro [4]. MSCs can be alternatives to chondrocytes, and revitalizing various 3D porous scaffolds with MSCs have demonstrated efficient regeneration of bone and cartilage [5,6]. MSCs are spindle-shaped and plastic-adherent cells. They can be harvested from various tissues capable of differentiation into at least three lineages: Osteoblasts, chondrocytes, and adipocytes. Their regenerative properties through the secretion of soluble factors contribute to their potential roles in regenerating several tissues and immunological tolerance [7]. Bone marrow mesenchymal stem cell (BMMSC) is a popular source of hMSC harvested through invasive methods. However, surgical complications and age-related reduction in self-renewal capacity have prompted researchers to seek alternative sources of hMSC [8,9]. Adipose-derived (ADMSC) and Wharton’s jelly-derived (UMSC) mesenchymal stem cells have been suggested as alternative sources. ADMSCs can be obtained from human lipoaspirates by a less invasive method [10]. Wharton’s jelly is a connective tissue in the human umbilical cord, and UMSCs can be isolated non-invasively from this tissue without ethical concerns. UMSCs are more primitive, proliferative, and immunosuppressive than adult hMSCs [11].

Revitalizing the microporous β-tricalcium phosphate (β-TCP) ceramics with autologous chondrocytes to repair osteochondral defects in sheep has been reported. Integration of newly formed cartilage into the surrounding native cartilage and completely restored structure of cancellous bone were found [12]. This intriguing osteochondral construct composed of a chondrogenic cell layer and β-TCP ceramics inspired us to evaluate the capability of hDCB to serve as a scaffold for osteogenesis of hMSCs but especially for the chondrogenesis of hMSCs in vitro.

Given that cell therapy requires a large number of cells, autologous and allogeneic MSCs have to be expanded to reach the amount needed for clinical purposes. According to a previous study, although stemness of late-passage hMSCs was reduced, they still had the potential for osteogenic and chondrogenic differentiation. They expressed surface markers CD105, CD73, and CD90 that meet the minimal standard criteria [13]. This study characterized late-passage hMSCs using flow cytometry and examined cell morphology and their potential to differentiate into osteogenic and chondrogenic lineages. The mechanical properties and pore sizes of hDCB were examined. The cytocompatibility of hDCB was evaluated using the MTT assay and immunostaining for F-actin. Osteogenic and chondrogenic potentials of hMSCs from different tissue origins (bone marrow, adipose tissue, and Wharton’s jelly) on hDCB were analyzed using immunocytochemistry, chemical stainings, and real-time quantitative PCR.

## 2. Results

### 2.1. Characterization of hMSCs and hDCB

**Figure 1:**
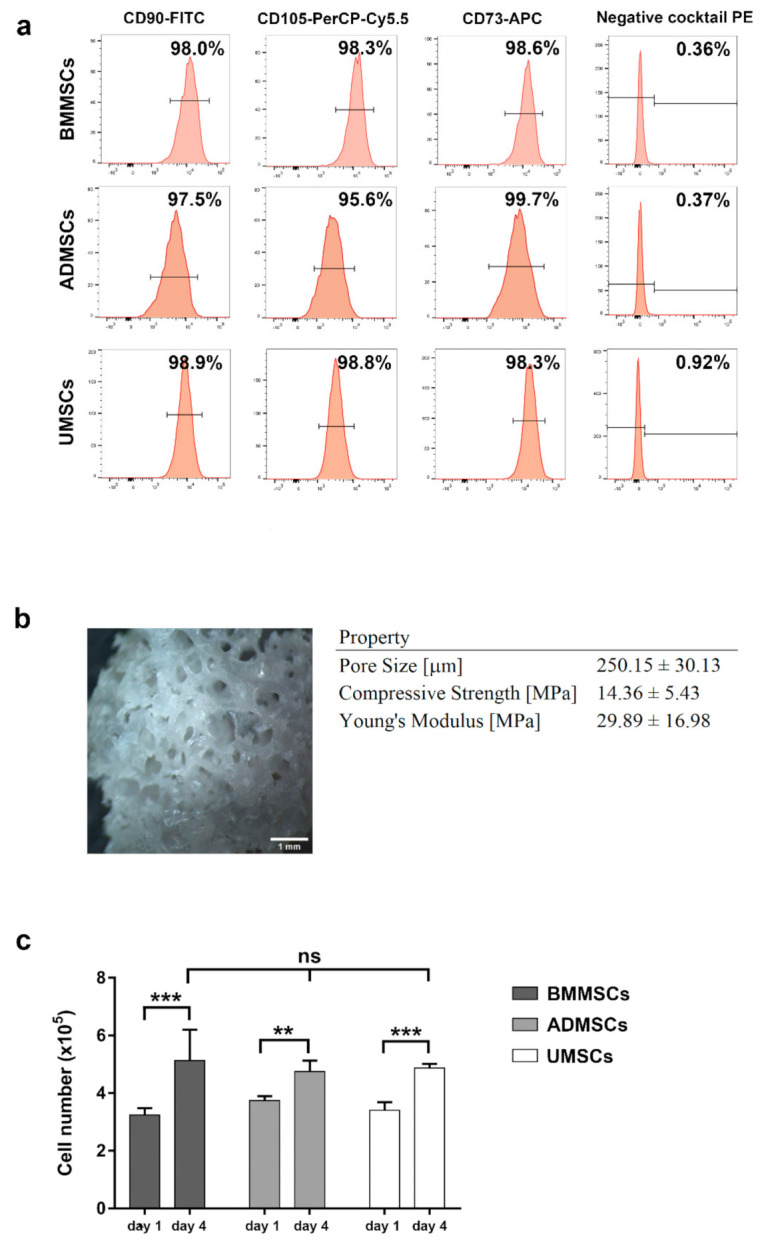
Characterization of hMSCs and hDCB blocks. (**a**) Flow cytometry analysis showed that the late-passage (P7–P10) hMSCs were positive for MSC surface markers (CD90, CD105, and CD73) but negative for hematopoietic markers (negative cocktail PE: CD11b, CD19, CD34, CD45, and HLA-DR). (**b**) Five hDCB blocks from each donor were used to measure the pore sizes and mechanical strength. Results showed the range of pore sizes and mechanical strength. (**c**) Cell viability assay of hMSCs on hDCB blocks was performed on days 1 and 4. Bars represent the mean ± standard error of the mean. A one-way ANOVA with Turkey’s post hoc test was used to examine the differences between experimental groups. Statistical significance is represented as ** *p* < 0.01, *** *p* < 0.001, and not significant (ns).

To confirm the identity of late-passage hMSCs that meet the minimal standard criteria, expression of MSC and hematopoietic markers was assessed by flow cytometry. As shown in Figure 1a, hMSCs derived from bone marrow, adipose tissue, and Wharton’s jelly expressed specific MSC markers (CD73, CD90, and CD105) but not hematopoietic markers, CD11b, CD19, CD34, CD45, and HLA-DR. The findings also showed a maintained spindle-like morphology and the osteogenic and chondrogenic potential of late-passage hMSCs (Figures S1 and S2). The pore sizes of hDCB blocks were 250.15 ± 30.13 μm. The compression strength and Young’s modulus of hDCB blocks were 14.36 ± 5.43 and 29.89 ± 16.98 MPa, respectively (Figure 1b).

### 2.2. Proliferation and Adhesion of hMSCs on hDCB Blocks

**Figure 2:**
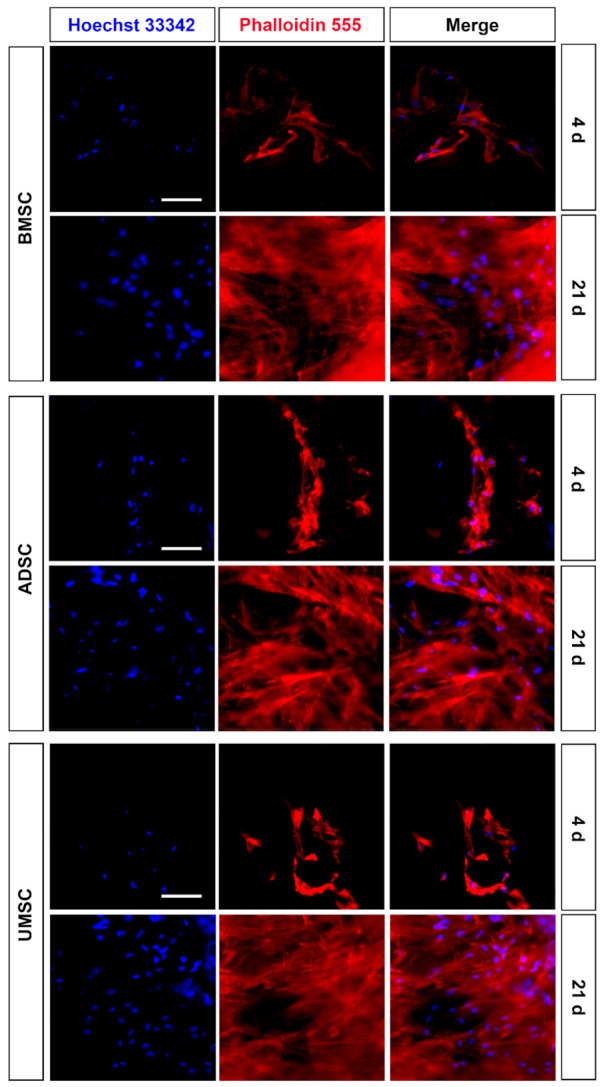
Identification of proliferation and adhesion of hMSCs on hDCB blocks. The left panels displayed nuclear staining, the middle panels showed F-actin staining, and the right panels displayed merged channels. F-actin and nuclei were stained with Phalloidin 555 (red) and Hoechst 33,342 (blue), respectively. Scale bars are 50 μm.

An MTT assay and immunocytochemical staining for F-actin were performed to assess cell viability and cell adhesion of hMSCs on hDCB blocks, respectively. Comparison of MTT assay results on days 1 and 4 indicated that hMSCs survived and proliferated significantly on hDCB blocks (1.58-, 1.27-, and 1.43-fold increases in BMMSCs, ADMSCs, and UMSCs, respectively). Furthermore, analysis of hMSC viability on day 4 indicated no significant difference in the number of cells among BMMSCs, ADMSCs, and UMSCs (Figure 1C). Immunocytochemical analysis of F-actin was performed on days 4 and 21 to visualize hMSC morphology on hDCB blocks and revealed that hMSCs grew and adhered well on hDCB blocks until day 21 (Figure 2).

### 2.3. Production of Osteogenic Proteins and Calcium Deposition of hMSCs on hDCB Blocks

**Figure 3:**
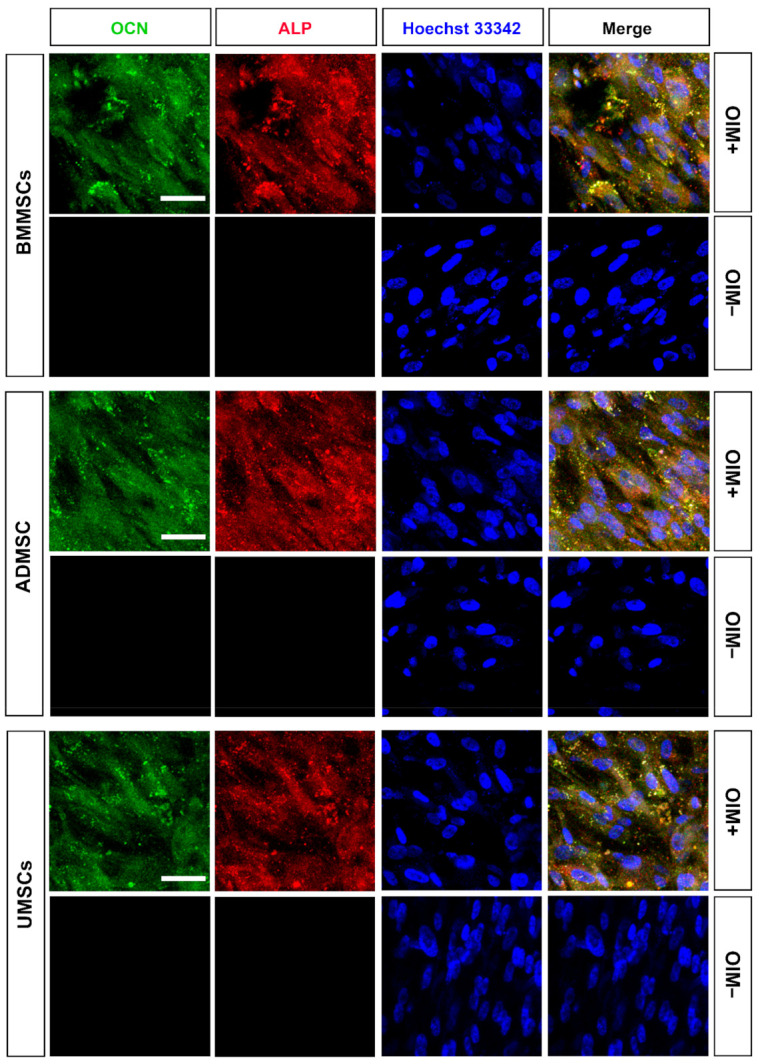
Characterization of osteogenesis of hMSCs on hDCB blocks. The hMSCs of three origins were seeded onto hDCB blocks, treated with or without an osteogenic differentiation medium for 14 days (OIM+ and OIM−, respectively), and then stained with osteocalcin (OCN) and alkaline phosphatase (ALP) antibodies to evaluate the expression of osteogenic proteins. Green, red, and blue represent OCN, ALP, and nuclei, respectively. Scale bars are 50 μm.

**Figure 4:**
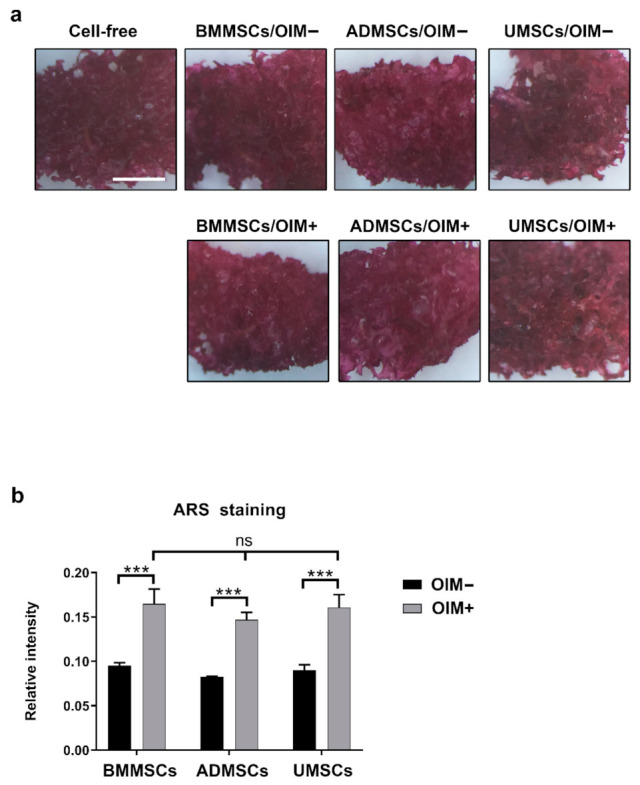
Identification of calcium deposition on hDCB blocks with hMSCs using Alizarin Red S staining. The hDCB blocks were seeded with hMSCs, and their osteogenic potential was analyzed after treatment with or without an osteogenic differentiation medium (OIM+ and OIM−, respectively) on day 14. (**a**) The production of bone ECM on cell-seeded hDCB blocks after osteoinduction was evaluated using Alizarin Red S staining. (**b**) The Alizarin Red S staining results were normalized to cell-free hDCB blocks. A one-way ANOVA with Turkey’s post hoc test was used to examine the differences between experimental groups. Statistical significance is represented as *** *p* < 0.001, and not significant (ns). Scale bars are 1 mm.

In vitro osteogenic induction was used to assess the osteogenic potential of BMMSCs, ADMSCs, and UMSCs. Immunocytochemical staining revealed the expression of osteogenic markers, namely OCN and ALP, after in vitro osteogenic induction of hMSCs on hDCB blocks (Figure 3). The penetration of cells into the deep layer of hDCB was also observed (Figure S3). Moreover, Alizarin Red S staining was performed. The staining results of Alizarin Red S were hard to distinguish (Figure 4a). Hence, the absorbance values of extracts from hDCB blocks were quantified. Consequently, the formation of a mineralized complex in osteoinduced hMSCs on hDCB blocks was increased compared with that in corresponding noninduced controls (1.74-, 1.77-, and 1.79-fold increase in BMMSCs, ADMSCs, and UMSCs, respectively), and no differences were observed among osteoinduced groups (Figure 4b).

### 2.4. Production of Chondrogenic Proteins and Proteoglycans of hMSCs on hDCB Blocks

**Figure 5:**
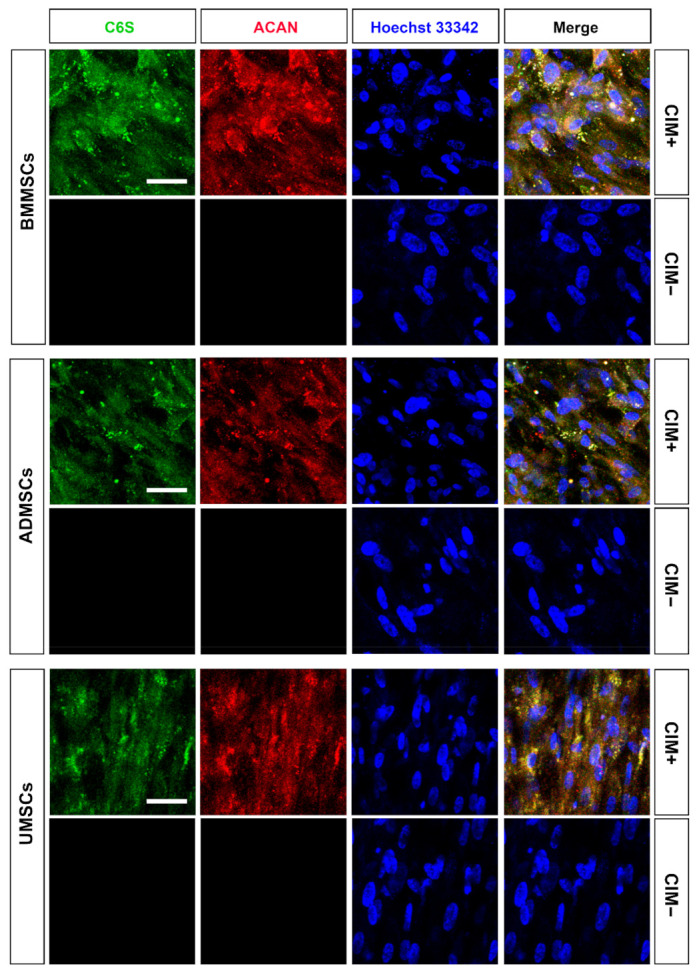
Characterization of chondrogenesis of hMSCs on hDCB blocks. The hMSCs of three origins were seeded onto hDCB blocks, treated with or without chondrogenic differentiation medium for 21 days (CIM+ and CIM−, respectively), stained with chondroitin 6–sulfate (C6S) and aggrecan (ACAN) antibodies to assess chondrogenesis. Green, red, and blue represent C6S, ACAN, and nuclei, respectively. Scale bars are 50 μm.

**Figure 6:**
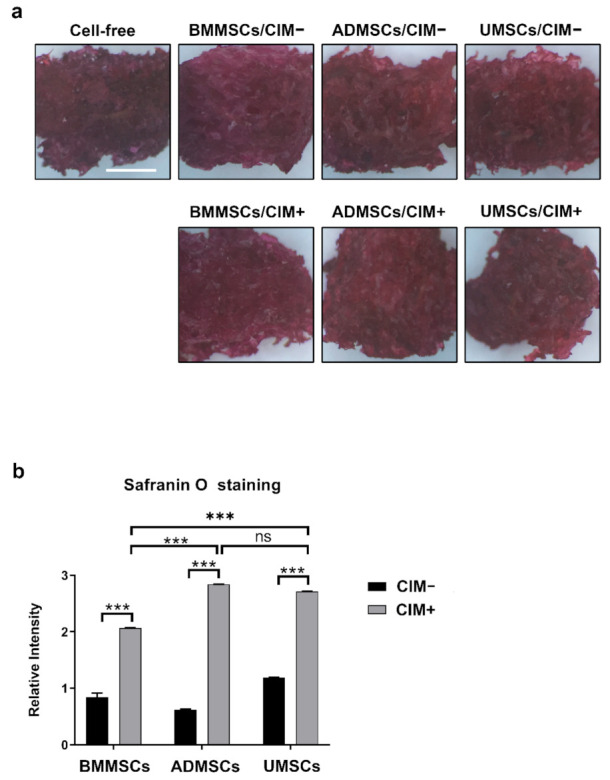
Quantification of proteoglycan on hDCB blocks with hMSCs using Safranin O staining. The hMSCs were seeded on hDCB blocks, and their chondrogenic ability was analyzed through treatment of hMSCs with or without chondrogenic differentiation medium (CIM+ and CIM−, respectively) on day 21. (**a**) The production of cartilage ECM on cell-seeded hDCB blocks after chondroinduction was assessed using Safranin O staining. Scale bars are 1 mm. (**b**) Results of Safranin O staining were normalized to cell-free hDCB blocks. A one-way ANOVA with Turkey’s post hoc test was used to examine the differences between experimental groups. Statistical significance is represented as *** *p* < 0.001, and not significant (ns).

Chondrogenic induction was carried out in vitro to evaluate the chondrogenic potential of BMMSCs, ADMSCs, and UMSCs. Chondrogenic markers (C6S and ACAN) were used for immunocytochemical analysis. Consequently, chondrogenic induction induced the production of C6S and ACAN in hMSCs on hDCB blocks (Figure 5). The staining further revealed that cells could penetrate into the deep layer of hDCB (Figure S4). To quantify their chondrogenic potential, Safranin O staining was performed for sulfated glycosaminoglycan. The absorbance values of extracts were determined since the staining results of Safranin O were hard to distinguish (Figure 6a). Findings showed that chondroinduced hMSCs produced more sulfated glycosaminoglycan than noninduced MSCs (2.44-, 4.56-, and 2.29-fold increase in BMMSCs, ADMSCs, and UMSCs, respectively). Among chondroinduced groups, ADMSCs and UMSCs produced more sulfated glycosaminoglycan than BMMSCs, and no difference was observed between ADMSCs and UMSCs (Figure 6b).

### 2.5. Comparison of Osteogenic and Chondrogenic Gene Expression Levels of hMSCs on hDCB Blocks Using Real-Time RT-qPCR

**Figure 7:**
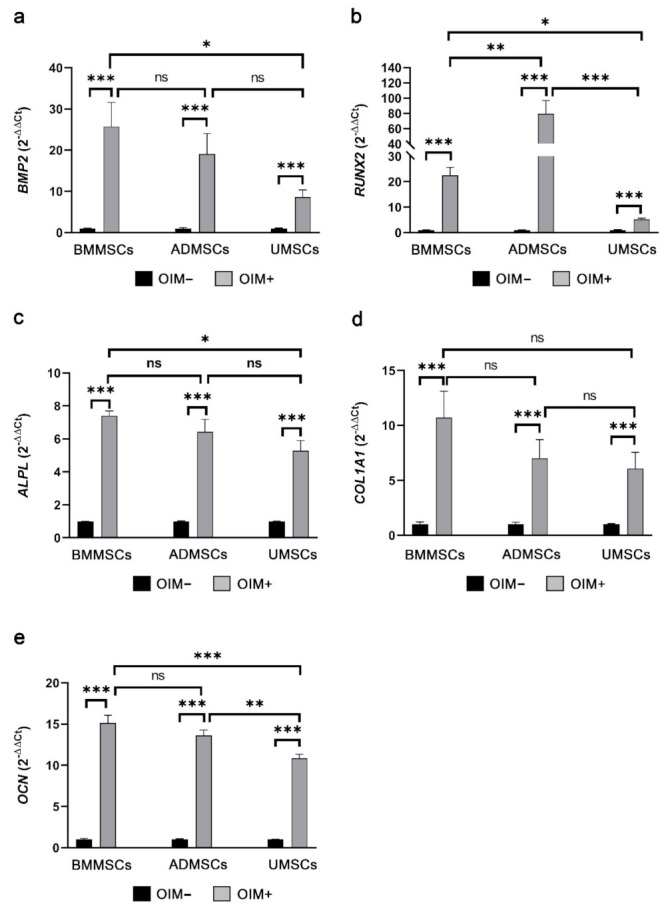
Real-time quantitative polymerase chain reaction analysis of osteogenic genes. Osteogenic genes *BMP2* (**a**), *RUNX2* (**b**), *ALPL* (**c**), *COL1A1* (**d**), and *OCN* (**e**) were analyzed, with *GAPDH* as the reference gene. Data derived from the treatment with an osteogenic differentiation medium (OIM+) were normalized to the noninduced (OIM−) groups. A one-way ANOVA with Turkey’s post hoc test was used to examine the differences between experimental groups. Bars indicate the mean ± standard error of the mean. Statistical significance is represented as * *p* < 0.05, ** *p* < 0.01, *** *p* < 0.001, and not significant (ns).

For osteogenesis, expression levels of *BMP2*, *RUNX2*, *ALPL*, *COL1A1*, and *OCN* were analyzed through RT-qPCR (primer pairs in Table 1). *BMP2* expression levels in osteoinduced BMMSCs, ADMSCs, and UMSCs were increased by 25.71-, 19.09-, and 8.66-fold compared with those in the noninduced groups (Figure 7a). The expression levels of *RUNX2* showed 22.53-, 79.53-, and 5.23-fold increase in osteoinduced BMMSCs, ADMSCs, and UMSCs compared with those in the noninduced groups (Figure 7b). *ALPL* expression levels were increased by 7.40-, 6.43-, and 5.28-fold in osteoinduced BMMSCs, ADMSCs, and UMSCs compared with those in the noninduced groups (Figure 7c). *COL1A1* expression levels were increased in osteoinduced BMMSCs, ADMSCs, and UMSCs by 10.70-, 7.02-, and 6.08-fold compared with those in the noninduced groups (Figure 7d). Expression levels of *OCN* were increased by 15.15-, 13.63-, and 10.85-fold compared with those in the noninduced groups (Figure 7e).

**Figure 8:**
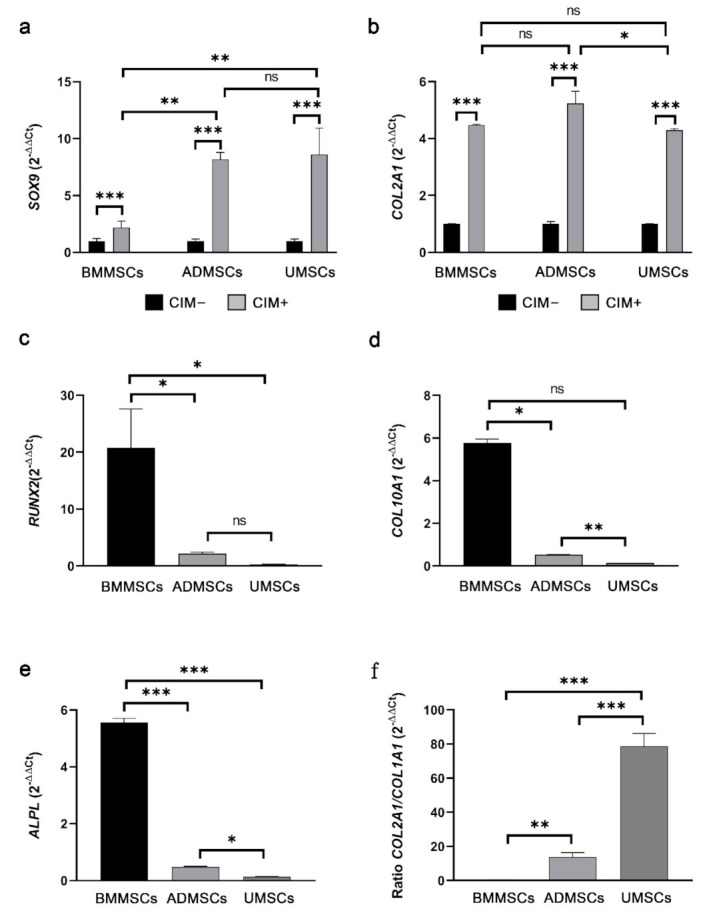
Real-time quantitative polymerase chain reaction analysis of chondrogenic differentiation. Chondrogenic genes (*SOX9* (**a**) and *COL2A1* (**b**)), hypertrophic differentiation genes (*RUNX2*(**c**), *COL10A1* (**d**), and *ALPL* (**e**)), and chondrocyte phenotype (ratio *COL2A1*/*COL1A1* (**f**)) were analyzed, with *GAPDH* used as the reference gene. Data derived from the treatment of MSCs with chondrogenic differentiation medium CIM+ were normalized to the noninduced (CIM–) groups. (**a**,**b**) Comparing the noninduced and chondroinduced groups. (**c**–**f**) Only showed the chondroinduced groups after being normalized to the noninduced groups. A one-way ANOVA with Turkey’s post hoc test was used to examine the differences between experimental groups. Bars indicate the mean ± standard error of the mean. Statistical significance is represented as * *p* < 0.05, ** *p* < 0.01, *** *p* < 0.001, and not significant (ns).

For chondrogenesis, *SOX9* and *COL2A1* expression levels were analyzed using RT-qPCR (primer pairs in Table 1). *SOX9* expression levels were increased by 2.20-, 8.18-, and 8.62-fold in chondroinduced BMMSCs, ADMSCs, and UMSCs compared with those in the noninduced groups (Figure 8a). *COL2A1* expression levels were increased by 4.48-, 5.23-, and 4.29-fold in chondroinduced BMMSCs, ADMSCs, and UMSCs compared with those in the noninduced groups (Figure 8b). The gene expression of hypertrophic differentiation (*RUNX2*, *COL10A1*, and *ALPL*) and chondrocyte phenotype (ratio *COL2A1/COL1A1*) in chondroinduced hMSCs were further analyzed. Results showed that the expression levels of *RUNX2, COL10A1,* and *ALPL* in chondroinduced BMMSCs were higher than those in chondroinduced ADMSCs and UMSCs (Figure 8c–e). The expression levels of the chondrocyte phenotype in chondroinduced BMMSCs were lower than those in chondroinduced ADMSCs and UMSCs. Moreover, UMSCs showed a better result of chondrocyte phenotype than ADMSCs (Figure 8f).

## 3. Discussion

This study demonstrated the different osteogenic and chondrogenic potential of BMMSCs, ADMSCs, and UMSCs on hDCB blocks. The present findings evidenced that hDCB blocks could be scaffolds for osteogenesis and chondrogenesis of hMSCs.

Since cell therapy requires a large number of cells, autologous or allogeneic hMSCs have to be expanded to reach the quantity needed for clinical purposes. Flow cytometry analysis was carried out to characterize their identity in this study. Results showed that late-passage hMSCs were positive for MSC markers but negative for hematopoietic markers. Moreover, late-passage hMSCs satisfied the minimal standard criteria of MSC [13]. Findings also showed the spindle-like morphology and the osteogenic and chondrogenic potential of late-passage hMSCs. Zhao et al. reported that late-passage hMSCs are suitable for laboratory and preclinical uses and can be scaled up to treat joint and bone diseases. In addition, Wall et al. found that calcium deposition reduced in passages 4 to 6 cells but increased to levels near or above the primary cells in passage 10 [14]. Although late-passage hMSCs could be used in the in vitro study, other potential concerns still needed to be addressed for regeneration applications. The reduced stemness, genomic instability, potential of lineage differentiation, and cell proliferation rate are major concerns for the application of MSCs [15]. If large numbers of cells are needed for clinical uses or tissue engineering, scaling up hMSCs in a 3D bioreactor could be a possible alternative [16].

Suitable microporosity and mechanical strength of scaffolds are essential for osteogenesis and chondrogenesis. It has been known that pore sizes ranging from 200–350 μm are preferred for osteoblast growth, cell aggregation, and endothelial cell proliferation [17,18,19]. Moreover, a negative linear relationship between porosity and compressive strength was proposed [20]. The scaffolds for chondrogenesis with pore sizes of 200 and 400 μm resulted in a higher expression of chondrogenic markers, SOX9 and COL2A1, and the best outcome of osteochondral repair has also been demonstrated [21]. Inconsistent mechanical strengths exist in both cancellous bone and cartilage due to their natural properties. For example, the compressive strength of trabecular bone ranges from 0.1–16 MPa, and Young’s modulus ranges from 50–500 MPa [22], while the compressive strength of natural cartilage ranges from 14–59 MPa, and Young’s modulus ranges from 12–15 MPa [23,24,25]. The present findings indicated that hDCB could be a suitable biomaterial for bone and cartilage tissue engineering due to its pore sizes (250.15 ± 30.13 μm) and mechanical properties (compressive strength: 14.36 ± 5.43 MPa; Young’s modulus: 29.89 ± 16.95 MPa).

Revitalizing scaffolds with MSCs have been a popular research field in regenerative medicine. We evaluated whether hDCB blocks could support cell growth using an MTT assay and immunofluorescence staining for F-actin. Results showed that hMSCs could grow on hDCB blocks indicating that hDCB blocks provided a natural microenvironment for cell growth. Furthermore, no differences in growth rate were observed among hMSCs. The present data are consistent with findings of Labutin et al., that thermal and γ-irradiated sterilization are favorable methods for promoting rat BMMSC survival on hDCB [26]. Furthermore, the present results for cell growth are concurrent with those of Heo et al., indicating no differences in proliferation potential of BMMSCs, ADMSCs, and UMSCs [27].

Osteogenic and chondrogenic markers in hMSCs on hDCB blocks are vital indicators of their successful induction. Some studies only performed chemical staining to assess the osteogenic and chondrogenic differentiation of MSCs. Other studies did not compare between noninduced and induced MSCs [28,29], which is crucial for reducing the risk of misleading results [30]. Here, we performed immunofluorescence and chemical staining simultaneously to assess the production of ECMs by hMSCs after osteogenic and chondrogenic induction. Immunofluorescence staining results showed that hMSCs could be successfully induced to differentiate into osteogenic cells (OCN and ALP) on hDCB blocks. The penetration of cells into the deep layer of hDCB was also observed. Herein, no differences among hMSCs were observed through Alizarin Red S staining. However, the RT-qPCR analysis revealed that the osteogenic potential of osteoinduced UMSCs was lower than the osteoinduced BMMSCs and ADMSCs. The present RT-qPCR analysis results showed almost the same expression of osteogenic genes in both osteoinduced BMMSCs and ADMSCs. Differential osteogenic potential was evident in many studies. Reports evidenced that osteogenic potential was dependent on materials, such as polycaprolactone-tricalcium-phosphate and bioactive glass [31,32]. Zhang et al. seeded BMMSCs and ADMSCs on a 3D scaffold and found higher levels of osteogenic genes expressed in BMMSCs. Rath et al. showed that ADMSCs seeded on bioglass-based scaffolds possessed higher osteogenic potential than BMMSCs. ADMSCs and UMSCs seeded on the nanocomposite scaffold have shown inferior osteogenesis compared with BMMSCs in rats with critical-size calvarial defects [33].

For chondrogenesis, immunofluorescence, Safranin O staining, and RT-qPCR analysis were performed to assess the chondrogenic potential of hMSCs. Findings showed that hMSCs could be induced to differentiate into chondrogenic cells (C6S and ACAN) on hDCB scaffolds. The staining revealed that cells could penetrate into the deep layer of hDCB. The results of Safranin O staining showed that chondroinduced ADMSCs and UMSCs produced more proteoglycan than chondroinduced BMMSCs. To understand the intrinsic gene expression, we performed RT-qPCR to evaluate the chondrogenic potential and found higher chondrogenic potential in ADMSCs and UMSCs than in BMMSCs. It has been reported that BMMSCs in hydrogel and hyaluronic acid scaffolds showed a higher potential of chondrogenesis in vitro compared with hMSCs from other tissue sources [34,35]. ADMSCs on a platelet-rich plasma gel scaffold resulted in better chondrogenesis in vivo compared with BMMSCs [36]. Comparing Wharton’s jelly MSCs and bone marrow MSCs showed that the expression levels of hypertrophic genes, *RUNX2* and *COL10*, were lower in Wharton’s jelly MSCs than in bone marrow MSCs [37], which is similar to our present finding. Previous results indicate that the chondrogenic potential of MSCs is dependent on several factors, such as the culture environment (normoxia or hypoxia) [38,39], growth factors (TGF-β1 or TGF-β3), 2D or 3D culture systems, and materials used [40,41]. Moreover, we found much higher expression of hypertrophy-related genes (*RUNX2, COL10A1, ALPL,* and ratio *COL2A1/COL1A1*) in BMMSCs on hDCB blocks. Prior studies have shown that hypertrophic MSCs underwent apoptosis and calcification, and cocultured BMMSCs constituted cartilage constructs that were prone to ossification and vascular invasion in vivo [42,43].

Using an osteochondral construct for osteochondral repair has been reported. A β-TCP scaffold seeded with chondrocytes showed the restoration of osteochondral bone and integration of newly formed cartilage into the surrounding native cartilage [12]. In the present study, cells were directly seeded onto the microporous hDCB blocks and induced to differentiate into chondrogenic cells. This osteochondral construct composed of a chondrogenic cell layer and a bony part of hDCB may have the potential for osteochondral regeneration. The hDCB blocks can provide enough mechanical strength, and the pore sizes of this material allow nutrients to be transported. Moreover, the production of ECM on this scaffold with chondroinduced hMSCs is evident in the present study. We propose that the microenvironment of osteochondral bone might influence cell fate determination of this osteochondral construct after implantation. That is, the side of the cell-seeded scaffold near the bone will connect to the surrounding bone tissue and undergo hypertrophy, then mineralizes and finally becomes bone. The side of the cell-seeded scaffold near the cartilage will integrate into the surrounding cartilage tissue. As mentioned above, such a hypothesis needs to be further validated using an animal model of osteochondral defects.

Taken together, results of this study demonstrate that the hDCB can serve as a scaffold for osteogenesis and chondrogenesis of hMSCs. Moreover, BMMSCs and ADMSCs possessed higher osteogenic potential than UMSCs. For chondrogenesis, ADMSCs and UMSCs are better than BMMSCs. The resulting osteochondral construct composed of a chondrogenic layer (chondrogenic hMSCs) and a bony layer (hDCB blocks) may potentially treat osteochondral defects.

## 4. Conclusions

The present study compared the osteogenic and chondrogenic potential of BMMSCs, ADMSCs, and UMSCs on hDCB blocks. Results indicate that hDCB blocks could be scaffolds for osteogenesis and chondrogenesis of hMSCs due to the natural microenvironment for hMSCs to grow and differentiate into osteogenic and chondrogenic lineages after induction. Findings also show that BMMSCs and ADMSCs are better than UMSCs in osteogenesis, but ADMSCs and UMSCs have more significant chondrogenic potential than BMMSCs. The natural properties of hDCB blocks render them a more suitable alternative for repairing bone defects when combined with hMSCs. Further in vivo studies of osteochondral defects using the osteochondral construct of chondrogenic hMSCs-seeded hDCB are needed.

## 5. Materials and Methods

### 5.1. Isolation and Expansion of Human MSCs

BMMSCs and ADMSCs were obtained from Dr. Jung-Pan Wang’s lab [44,45]. UMSCs were obtained from Professor Yu-Show Fu’s Lab [46]. Isolation of human BMMSCs, ADMSCs, and UMSCs followed procedures reported in previous studies [28,46,47]. The hMSC samples that had been thoroughly delinked from cohort members were obtained from the above labs. The hMSC phenotypes were characterized by the FACS CantoII Cytometer System running Diva software and using a human MSC analysis kit (562245, BD Company, Franklin Lakes, NJ, USA) [48]. The hMSCs at passages 7–10 were used due to the need for subsequent experiments, and the late-passage MSCs meet the minimal standard criteria [13] according to the flow cytometry analysis results. They positively expressed CD73, CD90, and CD105 MSC surface markers but negatively expressed hematopoietic markers, including CD11b, CD19, CD34, CD45, and HLA-DR. The hMSCs were seeded in 10-cm culture dishes and cultured in an α-modified Eagle’s medium (α-MEM; Thermo Fisher Scientific, Waltham, MA, USA) supplemented with 10% heat-inactivated fetal bovine serum (FBS; Thermo Fisher Scientific), penicillin (100 μg/mL), and streptomycin (100 μg/mL) and incubated at 37 °C in a 5% CO_2_ atmosphere. After cells reached 80–90% confluence, they were trypsinized and subsequently suspended in the growth medium for later use or stored in liquid nitrogen.

### 5.2. Processing of Allogeneic Bone

Distal femurs were harvested from two women who had undergone knee osteoarthritis replacement surgery (aged 75 and 77). The procedures were approved by the Institutional Review Board of Taipei Veterans General Hospital, and informed consent was obtained from the donors following the tenets of the Declaration of Helsinki. The harvested bone was examined for infection (hepatitis B virus, hepatitis C virus, human immunodeficiency virus, syphilis, and other infections). After removal of surrounding tissues, the distal femurs were cut into blocks (3 mm^3^) with a medical hand saw. The wash and γ-irradiation procedures followed those of a previous study [26]. Briefly, bone blocks were washed in distilled water three times for 60 min at 60 °C. The first wash cycle was performed in 10% sodium hydrocarbonate for 20 min, 15 min in hydrodynamic flow, sonication with 10% sodium hydrocarbonate for 20 min at 60 °C, hydrodynamic flow for 15 min, 10% sodium hydrocarbonate for 15 min at 1850× *g*, centrifugation for 15 min at 1850× *g*. The second wash cycle was conducted following the same procedures as the first wash with distilled water rather than sodium hydrocarbonate. This cycle was repeated five times. The third cycle was performed in 3% hydrogen peroxide at 60 °C in a shaking water bath, sonicator, and orbital shaker for 20 min each. Finally, bone blocks were washed in 70% ethanol three times for 60 min, then in distilled water three times for 15 min at 60 °C. The blocks were dried at 60 °C and frozen at −80 °C. The blocks were lyophilized for two days and packed for γ-irradiation (25 kGy). The packed sterilized cancellous bone blocks were stored at −80 °C.

### 5.3. Characterization of hDCB Block

The measurement of pore sizes was performed manually using dissecting microscopic images. The mechanical strength of hDCB blocks was measured according to a previous study [49]. The bone blocks were compressed at a rate of 3 mm/min at room temperature, and the compressive modulus was then calculated using the stress-strain curve and the sample dimensions. Five hDCB blocks from each donor were used to determine the pore sizes and mechanical strength.

### 5.4. Osteogenic and Chondrogenic Differentiation of hMSCs

The hMSCs were cultured in the growth medium (α-MEM) supplemented with 10% FBS, 100 μg/mL penicillin, and 100 μg/mL streptomycin. The osteogenic medium consists of α-MEM supplemented with 10% FBS, 100 μg/mL penicillin, 100 μg/mL streptomycin, 0.1 mM non-essential amino acids, 10 mM β-glycerol-2-phosphate (Sigma-Aldrich, St. Louis, MO, USA), 100 nM dexamethasone (Sigma-Aldrich), and 0.05 mM L-ascorbic acid (Sigma-Aldrich). The chondrogenic medium consists of DMEM supplemented with ITS (10 μg/mL insulin, 5.5 μg/mL transferrin, 5 ng/mL selenium, 0.5 mg/mL FBS, 4.7 μg/mL linoleic acid) (Sigma-Aldrich), 0.1 mM ascorbic acid 2-phosphate (Sigma-Aldrich), 100 nM dexamethasone, and 10 ng/mL TGF-β3 (R&D systems, Minneapolis, MN, USA). The sterilized hDCB blocks were placed in 24-well plates, incubated in the growth medium overnight, and aspirated before cell seeding. After the cells reached 80–90% confluence, they were trypsinized and subsequently suspended in the growth medium. The hMSCs were seeded by dropping the cell suspension homogeneously onto the three scaffolds from each donor at a seeding density of 1 × 10^6^ cells per scaffold. The seeded scaffolds were incubated for 2 h at 37 °C in 5% CO_2_ to allow cell attachment, after which 1.5 mL of growth medium was added to each well. After 24 h, the growth medium was replaced with 1.5 mL of differentiation medium. Cultures were maintained at 37 °C in 5% CO_2_ for 2 weeks (osteogenesis) or 3 weeks (chondrogenesis), and 1.5 mL of differentiation medium was replaced completely every 3 days. The subsequent experiments were repeated three times (technical repeats).

### 5.5. MTT Assay

Cell proliferation was evaluated at selected time points. A 3-(4,5-dimethylthiazolyl-2)-2,5-diphenyltetrazolium bromide (MTT, Sigma-Aldrich) assay was performed to quantify the hMSC viability on hDCB blocks. In brief, the cell-seeded hDCB blocks were washed in phosphate-buffered saline (PBS) before MTT assays were performed. They were then immersed in freshly prepared MTT reaction solution and incubated at 37 °C for 3 h in the dark. MTT was dissolved in DMSO, and absorbance was measured at 570 nm with a Tecan Sunrise spectrophotometer (Tecan, Männedorf, Switzerland). To generate a standard curve, hMSCs were serially diluted from 10^6^ to 10^3^ cells/mL, and the standard curve of absorbance against the number of cells/mL was plotted after the MTT assay. The average absorbance value of each hMSC-loaded group from triplicate experiments was determined, and the blanks were subtracted from the samples. The number of cells was determined according to the linear portion of the standard curve.

### 5.6. Immunofluorescence

The following experiments used the whole-mount samples. Cell-seeded hDCB blocks were fixed in 4% paraformaldehyde (PFA) for 20 min at room temperature. For F-actin staining, Phalloidin-iFluor 555 Reagent (ab176756, Abcam, Cambridge, UK) was applied at a 1:1000 dilution in 1% BSA with Hoechst 33,342 (ab228551, Abcam). For immunocytochemical analysis, 1% BSA was used as a blocking solution and incubated at 25 °C for 1 h after several washes in PBS. The bone blocks were then stained with the following primary antibodies (diluted to 1:250 in 1% BSA) in 1% BSA overnight at 4 °C: Mouse anti-osteocalcin (anti-OCN; SC-74495, Santa Cruz Biotechnology, Dallas, TX, USA) and rabbit anti-alkaline phosphatase (anti-ALP; GTX62596, GeneTex, Hsinchu City, Taiwan). The aggrecan core proteins and post-translational addition of chondroitin sulfate side chains were examined using rabbit anti-aggrecan (anti-ACAN; ab36861, Abcam, Cambridge, UK) and mouse anti–chondroitin 6–sulfate (anti-C6S; MAB2035, Merck, Darmstadt, Germany), respectively. Prior to using the anti-C6S antibody, the cell-seeded hDCB blocks were treated with chondroitinase AC overnight at 37 °C according to a previous study [50]. DyLight 488 (ab96875, Abcam) and DyLight 550 (ab96884, Abcam) fluorescence-conjugated secondary antibodies were used at a 1:200 dilution for 3 h at room temperature to visualize the expression of osteogenic and chondrogenic protein markers. Images were obtained using a Zeiss LSM 880 confocal microscope (Zeiss, Germany) with a 1-μm pinhole.

### 5.7. Alizarin Red S Staining

After osteogenic induction for 2 weeks, the cell-seeded hDCB blocks were fixed in 4% PFA for 20 min at room temperature and washed in PBS several times. The mineralized matrix generated herein from hMSCs was assessed through staining with 2% Alizarin Red S (Sigma-Aldrich) on hDCB blocks for 30 min and washed several times in PBS. Quantification was performed by dissolving the Alizarin Red S and calcium complex in a 10% cetylpyridinium chloride solution for 20 min. The optical density was subsequently measured at 550 nm with a Tecan Sunrise spectrophotometer (Tecan). Bone blocks without cells were used as background controls.

### 5.8. Safranin O Staining

At the end of chondroinduction, the cell-seeded hDCB blocks were fixed in 4% PFA for 20 min at room temperature and washed several times in PBS. The formation of acidic proteoglycans by hMSCs on hDCB blocks was analyzed through staining with 0.1% Safranin O (Sigma-Aldrich) for 30 min. After 30 min of reaction, isopropanol was used to wash out the absorbed Safranin O for 20 min. The absorbance was detected at 540 nm with a Tecan Sunrise spectrophotometer according to a previous study [51]. Bone blocks without cells were used as background controls.

### 5.9. Real-Time Quantitative Polymerase Chain Reaction Analysis of Osteogenic and Chondrogenic Genes

At the end of differentiation induction, samples were removed from culture and briefly rinsed with PBS. Total RNA was isolated using a TriRNA Pure Kit (Geneaid Biotech, New Taipei City, Taiwan), and cDNA was synthesized using an iScript^TM^ cDNA Synthesis Kit (Bio-Rad, Berkeley, CA, USA) according to the manufacturer’s instructions. A real-time quantitative polymerase chain reaction (RT-qPCR) was performed using a StepOne real-time PCR System (Thermo) with the Smart Quant Green Master Mix with ROX (Protech technology, Taipei, Taiwan), under the following cycling conditions: 95 °C for 10 min, followed by 40 cycles at 95 °C for 10 s and 60 °C for 30 s. The cycle threshold for each gene of interest was normalized against the housekeeping gene (*GAPDH*), and relative gene expression levels were determined using the 2^−ΔΔCt^ method compared with the corresponding noninduced group. The primer pairs of genes analyzed are listed in Table 1.

### 5.10. Statistical Analysis

GraphPad Prism v7.0 (GraphPad Software, San Diego, CA, USA) was used for statistical analysis. The one-way ANOVA with Turkey’s post hoc test was used to examine the differences between experimental groups. Significance was set at *p* < 0.05.

## Figures and Tables

**Table 1:** Primers for real-time quantitative polymerase chain reaction analysis.

Target Gene	Directions	Sequences	Accession No.	Product Size
*ALPL*	Forward	ACAGATGCCAACTTCCCACACG	NM_001200	112 bp
	Reverse	GCGGCAGACTTTGGTTTCTTGG		
*COL1A1*	Forward	TCCCCTCCACTCCTTCCCAAA	NM_000088	146 bp
	Reverse	GGCCACTTGGGTGTTTGAGCA		
*COL2A1*	Forward	TGGCTGACCTGACCTGATGTCC	NM_001844	95 bp
	Reverse	TGCAGTCTGCCCAGTTCAGGTC		
*COL10A1*	Forward	AGGCCCACTACCCAACACCAAGA	NM_000493	161 bp
	Reverse	CGTAGCCTGGTTTTCCTGGTGGTC		
*GAPDH*	Forward	TGAGCACCAGGTGGTCTCCTCTGAC	NM_001256799	147 bp
	Reverse	TCCACCACCCTGTTGCTGTAGCCA		
	Reverse	TGGGAGCAGCTGGGATGATG		
*RUNX2*	Forward	ATGACGTCCCCGTCCATCCA	NM_001024630	135 bp
	Reverse	GGAAGGCCAGAGGCAGAAGTCA		
*SOX9*	Forward	CCAAGCGCATTACCCACTTGTG	NM_000346	130 bp
	Reverse	CGATTCTCCATCATCCTCCACG		

## Data Availability

The data presented in this study are available on request from the corresponding author.

## Supplementary Materials

The following are available online at https://www.mdpi.com/article/10.3390/ijms22168987/s1.
