# Supplementary material for: Characterization of Osteogenesis and Chondrogenesis of Human Decellularized Allogeneic Bone with Mesenchymal Stem Cells Derived from Bone Marrow, Adipose Tissue, and Wharton’s Jelly
Source: Int J Mol Sci. 2021 Aug 20;22(16):8987. doi: 10.3390/ijms22168987 (PMC8396436; doi:10.3390/ijms22168987)
Supplement: Supplementary file 1 [file ijms-22-08987-s001.zip › ijms-1326813-supplementary.pdf]

Supplementary Materials

Alizarin Red S staining

Cells were seeded at 2×10^4 cells/cm^2 in culture dishes with the growth medium. Once the cells reached a confluence level, they were incubated in an osteogenic medium. After osteogenic induction for two weeks, cells were fixed in 4% PFA for 20 min at room temperature and washed in PBS several times. The mineralized matrix generated herein from hMSCs was assessed by staining with 2% Alizarin Red S (Sigma) on hDCB blocks for 30 min and washed several times in PBS. Images were taken using an inverted microscope.

Alcian Blue staining

Cells were seeded at 2×10^4 cells/cm^2 in culture dishes with the growth medium. Once the cells reached a confluence level, they were incubated in a chondrogenic medium. At the end of chondroinduction, cells were fixed in 4% PFA for 20 min at room temperature and washed several times in PBS. The formation of acidic proteoglycans by hMSCs on hDCB blocks was analyzed by staining with 1% Alcian Blue solution (Sigma) for 30 min. Images were taken using an inverted microscope.

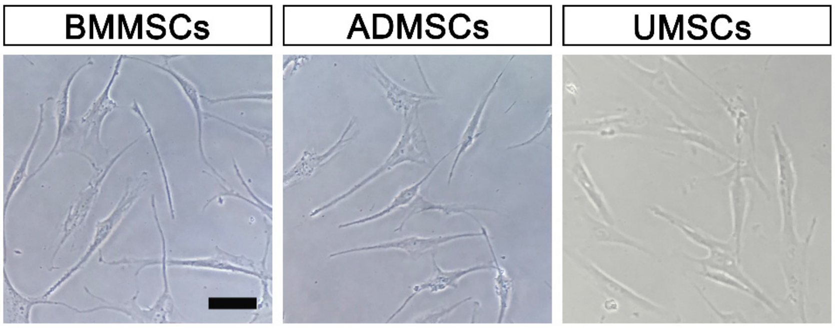

Figure S1. Cell morphology of BMMSCs, ADMSCs, and UMSCs. Morphologies of hMSCs at passage 10 were shown. Late-passage hMSCs maintained the spindle-like morphology. Scale bar is 100 μm.

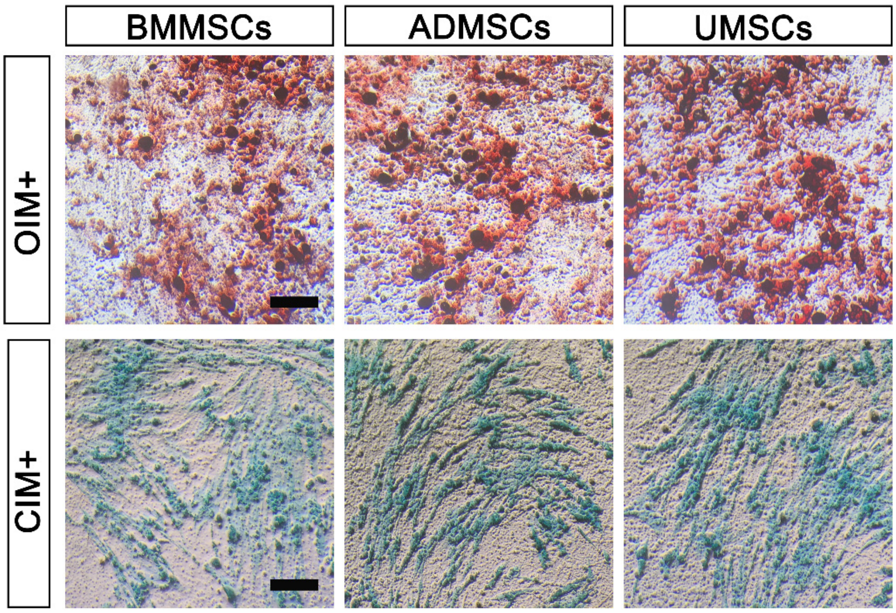

Figure S2. Osteogenesis and chondrogenesis of BMMSCs, ADMSCs, and UMSCs cultured in plates. Late-passage (P10) hMSCs were cultured in an osteogenic medium for 14 days and then stained with Alizarin Red S (upper panels). Late-passage (P10) hMSCs were cultured in a chondrogenic medium for 21 days and then stained with Alcian Blue (lower panels). Late-passage hMSCs could be induced to differentiate into osteogenic and chondrogenic lineages. Scale bars are 500 μm.

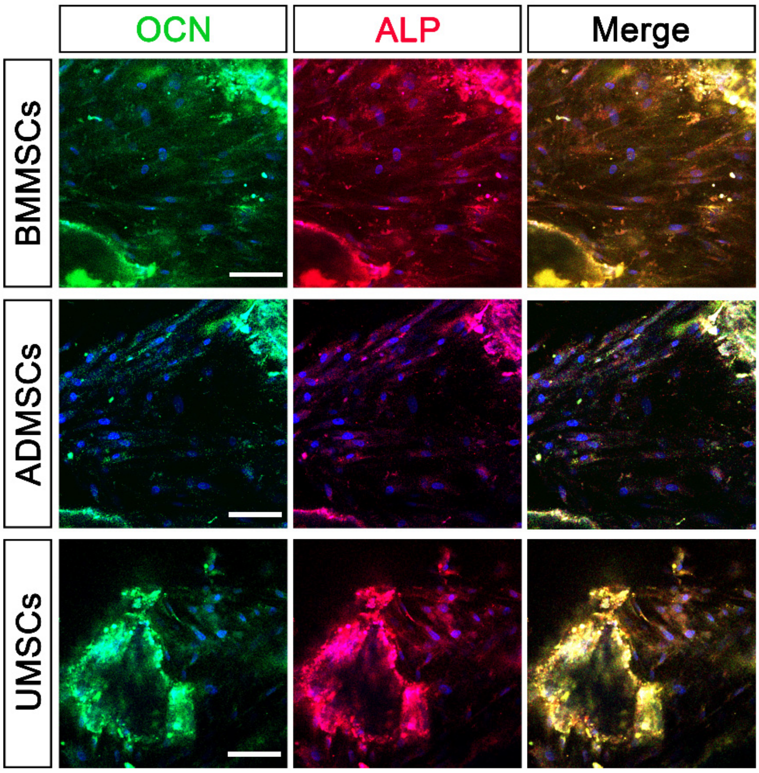

Figure S3. Immunocytochemistry staining of osteogenic markers on hDCB blocks with hMSCs after osteogenesis. BMMSCs, ADMSCs, and UMSCs on hDCB blocks were osteoinduced with

osteoinduction medium for 14 days and then stained with osteogenic markers, OCN and ALP. The deep layer near the pores of cell-seeded hDCBs showed positive stainings of OCN and ALP after osteogenesis and the cell morphology varied according to cell locations. Images were taken from one focal plane. Green, red, and blue represent the expression of OCN, ALP, and cell nuclei, respectively. Scale bars are 100 μm.

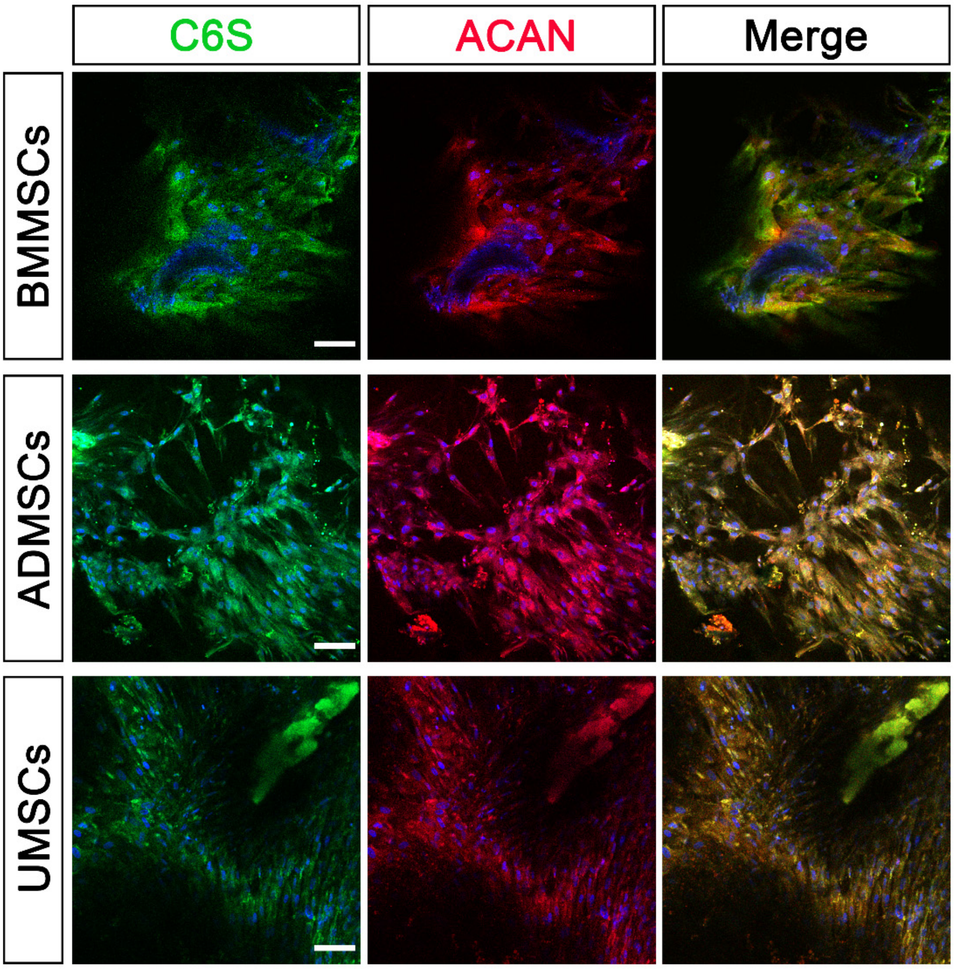

Figure S4. Immunocytochemistry staining of chondrogenic markers on hDCBs with hMSCs after chondrogenesis. BMMSCs, ADMSCs, and UMSCs on hDCB blocks were chondroinduced with chondroinduction medium for 21 days and stained with chondrogenic markers, C6S and ACAN. The deep layer near the pores of cell-seeded hDCBs showed positive stainings of C6S and ACAN after chondrogenesis and the cell morphology varied according to cell locations. Images were taken from one focal plane. Green, red, and blue represent the expression of C6S, ACAN, and cell nuclei, respectively. Scale bars are 100 μm.
